# Information Accessibility of the Charcoal Burning Suicide Method in Mainland China

**DOI:** 10.1371/journal.pone.0140686

**Published:** 2015-10-16

**Authors:** Qijin Cheng, Shu-Sen Chang, Yingqi Guo, Paul S. F. Yip

**Affiliations:** 1 HKJC Centre for Suicide Research and Prevention, The University of Hong Kong, Hong Kong SAR, China; 2 Institute of Health Behaviors and Community Sciences, and Department of Public Health, College of Public Health, National Taiwan University, Taipei, Taiwan; 3 Department of Social Work and Social Administration, The University of Hong Kong, Hong Kong SAR, China; Medical University of Vienna, AUSTRIA

## Abstract

**Background:**

There has been a marked rise in suicide by charcoal burning (CB) in some East Asian countries but little is known about its incidence in mainland China. We examined media-reported CB suicides and the availability of online information about the method in mainland China.

**Methods:**

We extracted and analyzed data for i) the characteristics and trends of fatal and nonfatal CB suicides reported by mainland Chinese newspapers (1998–2014); ii) trends and geographic variations in online searches using keywords relating to CB suicide (2011–2014); and iii) the content of Internet search results.

**Results:**

109 CB suicide attempts (89 fatal and 20 nonfatal) were reported by newspapers in 13 out of the 31 provinces or provincial-level-municipalities in mainland China. There were increasing trends in the incidence of reported CB suicides and in online searches using CB-related keywords. The province-level search intensities were correlated with CB suicide rates (Spearman’s correlation coefficient = 0.43 [95% confidence interval: 0.08–0.68]). Two-thirds of the web links retrieved using the search engine contained detailed information about the CB suicide method, of which 15% showed pro-suicide attitudes, and the majority (86%) did not encourage people to seek help.

**Limitations:**

The incidence of CB suicide was based on newspaper reports and likely to be underestimated.

**Conclusions:**

Mental health and suicide prevention professionals in mainland China should be alert to the increased use of this highly lethal suicide method. Better surveillance and intervention strategies need to be developed and implemented.

## Introduction

After charcoal burning (CB) suicide (i.e. suicide from carbon monoxide poisoning by inhaling barbecue charcoal gas) was first widely publicized in Hong Kong in November 1998, suicides using this method quickly became the second most common suicide method in Hong Kong and Taiwan within five years. Increases were also seen in Japan, South Korea, Singapore, and even countries in other parts of the world [1–3]. Over the last decade, this method has accounted for more than one thousand deaths in Taiwan and Hong Kong each year [3]. Furthermore, the method has been shown to have limited substitutions of other methods and it has an impact on overall suicide trends in some of these countries [3, 4]. Thus, it is important to monitor and prevent the further spread of the CB suicide method to regions where its use is still not that common.

Given the rapid spread of the method in the East Asian area and the geographic and cultural closeness of mainland China, Hong Kong, and Taiwan, it is reasonable to be concerned about whether it would spread to mainland China, where 120,000 suicides occur annually [5]. A recent study found that there might be a cross-regional spreading of new suicide methods via the Internet [6], and the number of Internet users in China has increased very rapidly in recent years, reaching around 500 million in 2011 [7]. Forensic studies reported individual cases of CB suicides in some mainland Chinese cities [8–10]. However, there is limited data available about both the incidence of CB suicide and the accessibility of information about it in mainland China in general. Challenges for investigating this topic include the limitation of the accuracy and coverage of the China national death registry system and the availability of reliable suicide data.

To better understand CB suicide in mainland China, this study investigates the accessibility of information about it by examining media-reported cases, online search trends, and online information. It also considers media representations of CB suicide as a trend proxy for CB suicide in the community, or at least to show the tip of the iceberg. Research in Hong Kong, Taiwan, and Japan has shown that media reports of, or online searches about, a new suicide method are significantly correlated with the incidence of suicides using it [1, 11–14]. Previous in-depth interviews with suicide attempters also suggested that information about new suicide methods in traditional media and/or on the internet is a crucial source of cognitive access to the means and may lead to increased suicide incidence [15–17].

The specific aims of this study are to examine: 1) to what extent CB suicide has been reported by the mass media in China; 2) to what extent the CB suicide method has been searched for online; and 3) to what extent detailed information about the CB suicide method is available on the Internet.

## Methods

We extracted and reviewed data from both traditional newspapers and online platforms. All the data are available in the public domain and aggregated at the population level so there are no privacy issues. We also extracted provincial population figures in mainland China (31 area units in total, including 27 provinces and 4 provincial-level municipalities, in mainland China) from the latest census report [18], and the number of provincial Internet users in mainland China from the China Internet Network Information Center (CNNIC) [19]. All the statistical analyses were carried out using SAS^®^ 9.4.

### Newspaper reports

We used the general term “charcoal burning suicide” (*Shao Tan Zi Sha* in Chinese pinyin) to search news reports of fatal and nonfatal suicide attempts using the method in mainland Chinese newspapers via the WiseNews database. A fatal suicide was defined as one where, according to the report, the person was found dead at the scene or died soon after receiving emergency treatment, and the cause of death was reported as a suicide. A nonfatal attempt was defined as one where the report indicated that the person had tried to kill him-/herself but did not die before it was published. The search period was set from November 1, 1998, which is just before the occurrence of the first widely publicized CB suicide in Hong Kong [2, 3], to December 31, 2014. All 335 mainland Chinese newspapers included in the WiseNews database were screened. Only cases from mainland China were included. We extracted the demographic and social characteristics of those cases as reported by the newspapers, including the person’s age, gender, the location of the event, the province in which the case had taken place, and whether the incident was a suicide pact (that is, a situation where two or more individuals had jointly attempted suicide). The differences in demographic and social characteristics between fatal and nonfatal attempts were examined using a chi-square test, or Fisher’s exact test where appropriate.

Time trends in fatal cases, and fatal and nonfatal cases combined, were investigated using a simple linear regression model. A time variable of 678 weeks, from the first week of 2002 to the last week of 2014, was included as an independent variable, and the number of cases reported in each week as a dependent variable in the models. The reporting date of each case was defined as the date on which the first news article about it was published. Cook’s distance (i.e. Cook’s D statistic) was used to evaluate the potential influential points. We also conducted sensitivity analyses by excluding major peak values.

### Online search trends

The amount of searches for “charcoal burning suicide” (*Shao Tan Zi Sha*) were extracted from the Baidu Index (http://index.baidu.com/), which is provided by Baidu for open access. Baidu is the most popular search engine in mainland China, and accounts for about 80% market share in China [20]. The earliest date when such data became available is January 1, 2011. Hence, we extracted weekly data from the first week of 2011 to the last week of 2014 at the national level. In addition, to investigate regional differences in search activity, we extracted the average search index for “charcoal burning suicide” in all 31 provinces or provincial-level municipalities in mainland China.

The national internet search trends for CB suicide were investigated using the same method described above for analyzing time trend in cases reported by the newspapers. A time variable of 209 weeks, from the first week of 2011 to the last week of 2014, was included as an independent variable, and the Baidu search activity per week as a dependent variable in the models.

When entering “CB suicide” into Baidu’s search box, the system would automatically recommend more keywords to help users refine their search queries. The recommendations are shown on the top of the second page of the search results and easily attract users’ attention. Therefore, we also included those recommended keywords, and repeated the same procedure until no new keywords relating to the CB suicide were shown in the recommendation box. Eventually, eight relevant keywords were identified, namely, “charcoal burning” (*Shao Tan*), “charcoal burning suicide method” (*Shao Tan Zi Sha Fang Fa*), “charcoal burning suicide correct method” (*Shao Tan Zi Sha Zheng Que Fang Fa*), “charcoal burning suicide painful” (*Shao Tan Zi Sha Tong Ku*), “is charcoal burning suicide painful” (*Shao Tan Zi Sha Tong Ku Ma*), “how much time charcoal burning suicide takes” (*Shao Tan Zi Sha Duo Shao Shi Jian*), “charcoal burning suicide success rate” (*Shao Tan Zi Sha Cheng Gong Lv*), and “live broadcast charcoal burning suicide” (*Zhi Bo Shao Tan Zi Sha*). Among the eight additional keywords, Baidu Index only provided open access to the search index data of three keywords, namely “charcoal burning,” “is charcoal burning suicide painful,” and “live broadcast charcoal burning suicide.” To test the sensitivity of our trend analysis, we replicated the analysis for these three additional keywords.

In addition, we examined the correlation between the average rate of publicized CB suicide per year and the average rate of search activities on “CB suicide” per year at the provincial level using Spearman’s correlation coefficient. The rate of publicized CB suicide was calculated as the mean annual number of CB suicides reported by the media in 2011–2014 divided by the population of each province. The rate of Internet search activity was calculated as the average search index of “CB suicide” (2011–2014) divided by the total number of Internet users in each province. To visualize the results, we produced two maps of mainland China to demonstrate the average rate of publicized CB suicide and the average rate of search activities on “CB suicide” respectively. "The shapefile map of Chinese provinces was obtained from the National Geomatics Center of China. ArcGIS version 10.2 was used to produce the maps. The Jenks Natural Break method was used to group all of the 31 provinces or provincial-level municipalities into 5 classes, which allows provinces in each class to have similar values and differences between classes to be maximized [21].

### Content analysis of online information

To assess the extent to which an online user could be exposed to detailed information about the CB suicide method, we conducted a content analysis on the Baidu search results retrieved using “charcoal burning suicide” and the eight other related keywords. Following previous work on online search results, we only included the results displayed on the first three pages in the analysis, because users are not likely to click through any further [22, 23]. Normally, 10 search results are displayed on each page. However, Baidu searches sometimes display a few sub-links under a search result, which are usually related to the original result. We also included these sub-links in our analysis. A total of 292 search results were downloaded from the 9 keywords. We clicked on all 292 results and reviewed the content on the linked webpages.

The content analysis focused on 1) whether or not a webpage described or visualized detailed information about the CB suicide method; 2) the overall attitude toward suicide displayed on the page (pro, anti, neutral, or mixed); and 3) whether or not the page encouraged people to seek help when feeling suicidal. “Detailed information about the CB suicide method” was defined as covering the types of materials needed and the environment where their use could lead to death. By “visualizing,” we denoted webpages that contained videos or photographs/graphics of the method. Such a coding protocol has been used in previous studies examining suicide-related content in printed media or on the Internet [22–24]. In addition, we grouped the information sources of the webpages into three types: 1) news media, which indicated that the content of a webpage was a news report (including textual, graphic, and video reports) published on a news website or portal website; 2) user-generated, which indicated that the page contained user-generated content, such as online forums, discussion platforms, and personal blogs; and 3) knowledge hubs, including wikipedia-style webpages and databases of academic publications. Only two search results were academic publications. The coding exercise was performed by the first author. The results were descriptively summarized. Differences in the website contents among the three types of information sources were examined using Fisher’s exact test.

## Results

### Newspaper reports

170 newspaper articles covering suicides or suicide attempts using the CB method were identified, reporting on 109 individuals who had attempted to end their lives (89 were fatal and 20 were nonfatal). As shown in Table 1, the majority of those reported cases were male (67.9%), aged below 30 (69.7%), occurred at a hotel (54.1%) and in Guangdong Province (54.1%), and involved suicide pacts (59.6%). There were no differences between fatal and nonfatal attempts with regard to gender, incidence location, incidence place, and whether or not a suicide pact was involved, but there was a difference in age. Information of age was more likely to be missed in nonfatal than fatal incidents reported by newspapers (30% vs 2%).

**Table 1:** Characteristics of fatal and nonfatal CB suicide attempts reported in mainland Chinese newspapers and the statistical differences between them.

*Characteristics*		Total (N = 109)	Fatal attempts (N = 89)	Nonfatal attempts (N = 20)	Fatal vs. nonfatal attempts		
		n (%)	n (%)	n (%)	χ^2^	df	*p*
***Gender***	***Female***	35 (32.1)	28 (31.5)	7 (35.0)	0.094	1	0.759
	***Male***	74 (67.9)	61 (68.5)	13 (65.0)			
***Age***	***Under 30*** **	76 (69.7)	65 (73.0)	11 (55.0)			0.002*
	***30~60***	23 (21.1)	20 (22.5)	3 (15.0)			
	***Above 60***	2 (1.8)	2 (2.2)	0 (0)			
	***Unknown***	8 (7.3)	2 (2.2)	6 (30.0)			
***Incidence location***	***Home or rental apt or dorm***	45 (41.3)	41 (46.1)	4 (20.0)			0.079*
	***Hotel***	59 (54.1)	43 (48.3)	16 (80.0)			
	***Office***	1 (0.9)	1 (1.1)	0 (0)			
	***Car***	4 (3.7)	4 (4.5)	0 (0)			
***Incidence province***	***Guangdong Province***	59 (54.1)	50 (56.2)	9 (45.0)	0.822	1	0.365
	***Others***	50 (45.9)	39 (43.8)	11 (55.0)			
***Suicide pact***	***Yes***	65 (59.6)	52 (58.4)	13 (65.0)	0.293	1	0.588
	***No***	44 (40.4)	37 (41.6)	7 (35.0)			

* *p* value of Fisher’s exact test.

** includes those where no age was reported but individual referred as a “youth” or “university student.”

CB suicide attempts were reported in 13 provinces or provincial-level municipalities. More than half (59/109; 54.1%) of the reported CB cases occurred in Guangdong Province, which borders Hong Kong; the rest of CB cases mostly occurred in provinces along the east coast, such as Zhejiang and Shanghai, while cases were also reported in inland provinces such as Sichuan and Gansu. The earliest reported case involved a young man in his twenties who killed himself in Guangzhou, the capital of Guangdong Province. The news report was published on February 9, 2002. Of note, the second case was a man originally from Hong Kong who died at his home in Shenzhen in 2005, and the third was a man originally from Taiwan who died at his home in Beijing in 2006.

It is also noteworthy that around 60% of the reported cases involved suicide pacts, in which two or more individuals intend to die together by burning charcoal (Table 1). In total, 23 suicide pacts, involving 65 individuals, were reported. 13 suicide pacts, involving 40 individuals, were formed by strangers who first met through online platforms. The case that accounted for the highest number of news reports was a pact in Shanghai involving three deaths (a couple and their adult child). This case was reported in 29 articles from 21 newspapers, circulating in different regions, starting on June 5, 2014 and continuing for a few days. The family’s suicide note indicated that they were unable to repay credit card debts of 2.4 million yuan (about US$392,000) [25].

**Fig 1:**
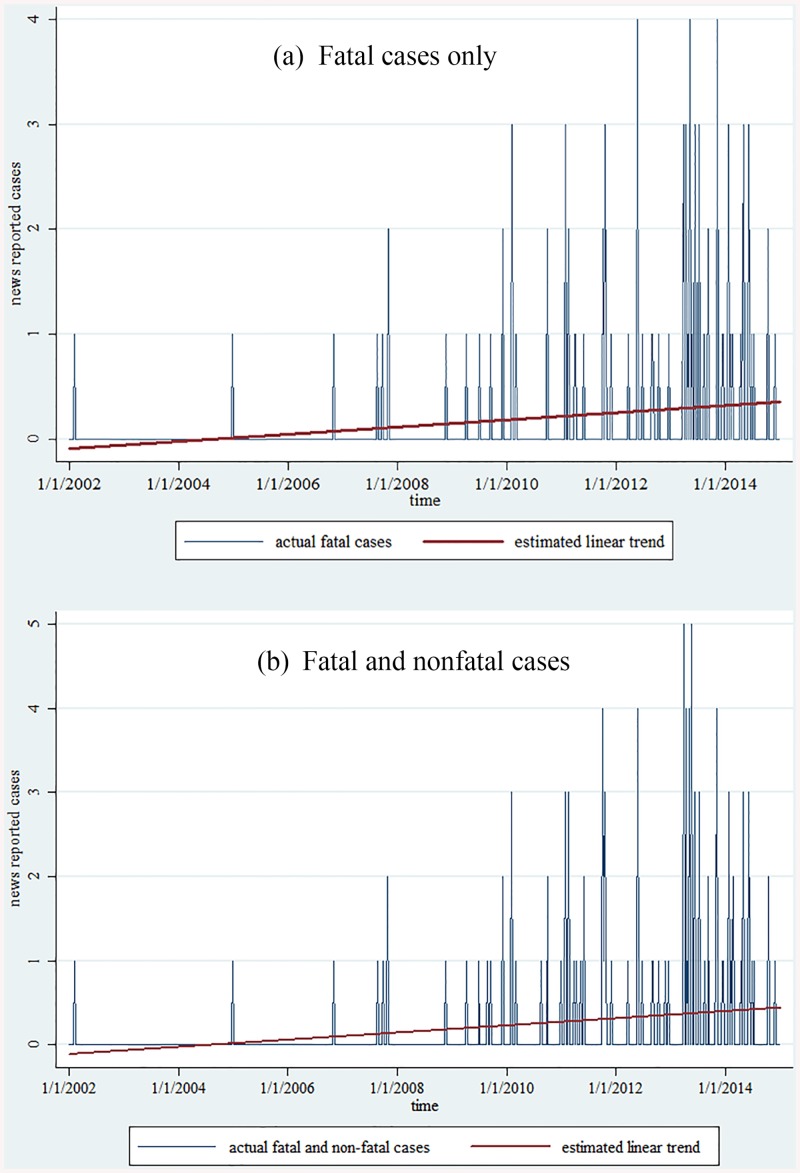
Trends in newspaper reports of fatal and nonfatal charcoal burning suicides.

Time trend analysis indicated an increase in CB cases reported in the newspapers, whether only fatal or both fatal and nonfatal cases were included (see Fig 1a and 1b). The coefficient of the simple linear regression of fatal cases was 0.0000939 (*p*<0.001) with 21 peaks (Fig 1a), whereas that of fatal and nonfatal cases was 0.0001164 (p<0.001) with 24 peaks (Fig 1b). After excluding major influential values, the increasing trends became 0.0000325 and 0.0000373 respectively, but remained statistically significant (*p*<0.001).

### Online searches

**Fig 2:**
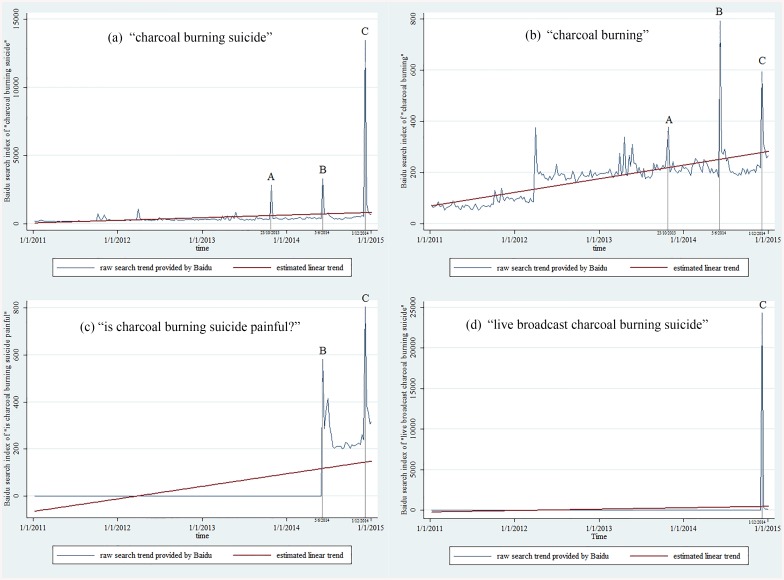
Baidu search activity for “charcoal burning suicide,” “charcoal burning,” “is charcoal burning suicide painful,” and “live broadcast charcoal burning suicide,” and their estimated trends. A: A 19-year-old man attempted suicide by burning charcoal in July 2013 and then tried again by overdosing in October 2013. The case was reported by a provincial newspaper on October 23, 2013 and the article was then reposted on many other news websites. B: A suicide pact with 3 deaths, including a couple in their 60s and their adult son, was reported by various newspapers on June 5, 2014 and the following few days. C: A 19-year-old man broadcast his charcoal burning suicide live on Sina Weibo (a social media website) and received over 20, 000 comments from Weibo users in about 10 hours. He was later found dead. This tragedy was reported by various media outlets on December 1, 2014 and the following few days.

According to the Baidu Index, search activity on “charcoal burning suicide” and “charcoal burning” could be traced back to the first week of 2011. Simple linear regression analysis of search activity on “charcoal burning suicide” showed an upward trend (regression coefficient = 0.531; *p* = 0.001) (Fig 2a). Cook’s distances suggested three influential points which coincided with three widely reported cases (see Fig 2a and its note). After excluding major peak values, the increasing trend became lesser, with a coefficient of 0.229, but remained statistically significant (*p*<0.001).

The search index of “is charcoal burning suicide painful” only started in the first week of June 2014, coinciding with a suicide pact that was widely reported by the newspapers (see details in the note to Fig 2). The index of “live broadcast charcoal burning suicide” only started in the first week of December 2014, coinciding with a suicide incidence where a young man broadcast himself on a popular social media platform about the process of killing himself using the CB method ([26], also see details in the note of Fig 2). These results suggest that the two additional search keywords did not become frequently used until particular incidents had become media events.

For the keyword “charcoal burning,” the coefficient of the simple linear regression was 0.146 (*p*<0.001), which suggests a slightly increasing trend compared with “charcoal burning suicide” (Fig 2b). The results of the Cook’s distance showed four influential points for the regression model, three of which were consistent with the three peaks for “charcoal burning suicide.” After excluding major influential values, the regression. The search term “is charcoal burning suicide painful” had a slightly increasing trend (coefficients of the simple linear regression = 0.146, *p*<0.001), whereas the term “live broadcast charcoal burning suicide” showed no clear trend (coefficient = 0.471, *p* = 0.087). After excluding major influential values, both keywords showed a small but significant increasing trend with coefficients of 0.091 (*p*<0.001) and 0.015 (*p* = 0.002) respectively (see Fig 2c and 2d). These results showed that online search activities related to CB suicide were generally increasing in mainland China, and the keyword “charcoal burning suicide” could be more sensitive and observable for monitoring online search trends than other keywords.

**Fig 3:**
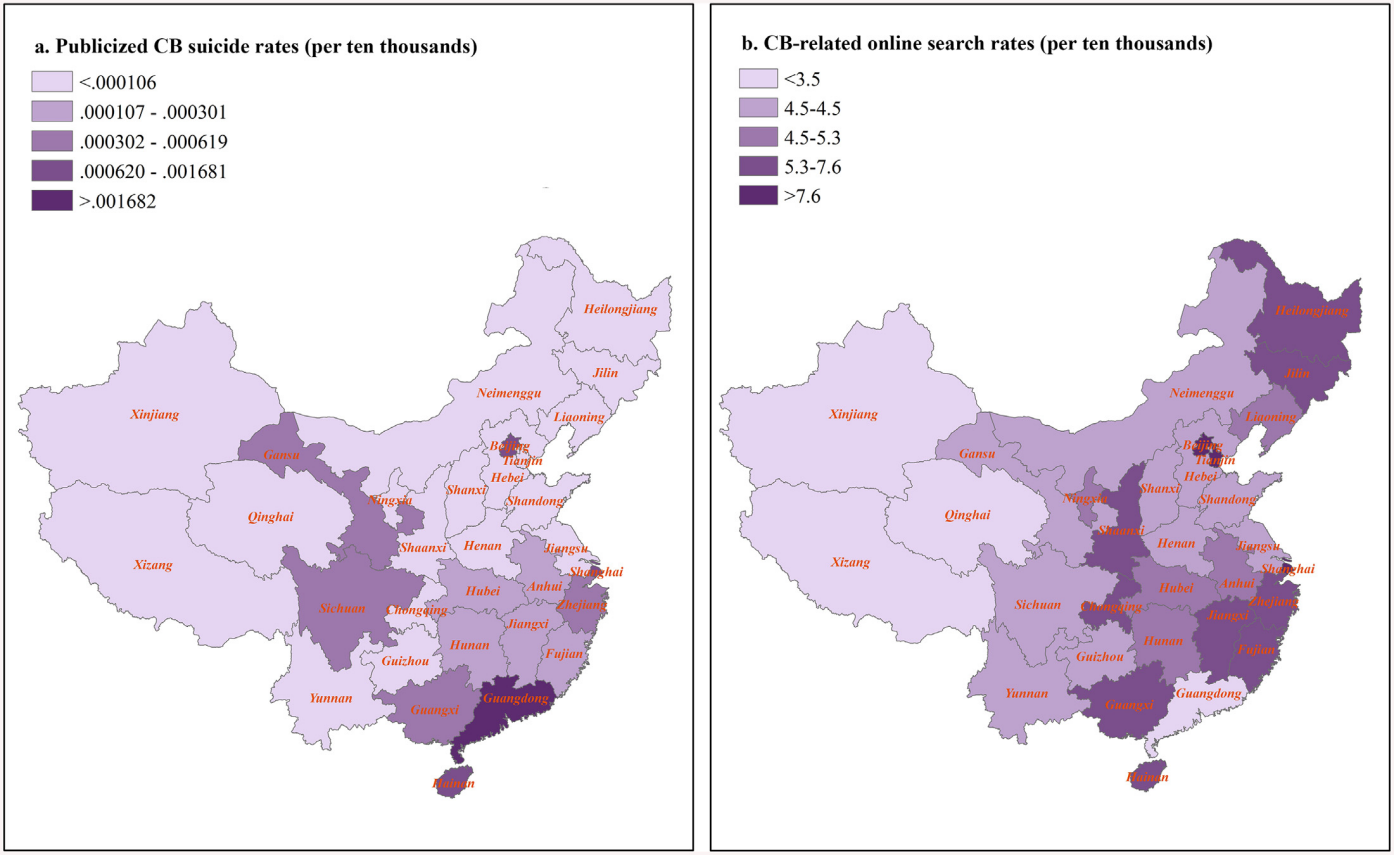
Maps of publicized CB suicide rates and CB-related online search rates across 31 provinces and provincial-level municipalities in mainland China (2011–2014).

The Spearman’s correlation coefficient between internet search rates and publicized CB suicide rates was 0.43 (95% CI 0.08 to 0.68), indicating that provinces with higher rates of publicized CB suicide also tend to have higher internet search rates. Fig 3a shows that the rate of publicized CB suicide is highest in Guangdong Province, Beijing, and Shanghai. Beijing and Shanghai are the two biggest cities in mainland China, where national media outlets cluster largely. Guangdong Province is bordered with Hong Kong SAR and also has an energetic market of metropolitan newspapers. Fig 3b shows that the internet search rates were highest in the three provincial-level municipalities, namely Tianjin, Beijing, and Shanghai. However, the search rate was relatively low in Guangdong, diluted by its massive number of internet users (see details in Table 2).

**Table 2:** Numbers and rates of CB-related online searches and publicized CB suicides across 31 provinces and provincial-level municipalities in mainland China (2011–2014).

					Online search rate vs. publicized CB suicide rate	
*31 Provinces and provincial-level municipalities (Descending by average search rate)*	Average Baidu search index of “CB suicide” (A)	Number of Internet users (10,000s) (B)	Number of CB suicide deaths reported by the media per year (C)	Population (10,000s) (D)	Average search rate (per million internet users) (= A/B)	Average publicized CB suicide rate (per million population) (= C/D)
***Tianjin***	45	866	0	1413	5.2	0
***Beijing***	79	1556	0.5	2069	5.08	0.025
***Shanghai***	74	1683	2.25	2380	4.4	0.095
***Hainan***	14	411	0.25	887	3.41	0.0275
***Fujian***	71	2402	0.75	3748	2.96	0.02
***Jilin***	33	1163	0	2750	2.84	0
***Jiangxi***	40	1468	0.25	4504	2.72	0.005
***Chongqing***	35	1293	0	2945	2.71	0
***Zhejiang***	90	3330	0.75	5477	2.7	0.0125
***Guangxi***	47	1774	0.25	4682	2.65	0.005
***Shaanxi***	44	1689	0	3753	2.61	0
***Hubei***	63	2491	0.25	5779	2.53	0.005
***Heilongjiang***	38	1514	0	3834	2.51	0
***Anhui***	51	2150	0.75	5988	2.37	0.0125
***Hunan***	57	2410	0.5	6639	2.37	0.0075
***Liaoning***	55	2453	0	4389	2.24	0
***Ningxia***	6	283	0	647	2.12	0
***Sichuan***	60	2835	1.25	8076	2.12	0.015
***Gansu***	19	894	0.25	2578	2.12	0.01
***Shanxi***	37	1755	0	3611	2.11	0
***Jiangsu***	83	4095	0	7920	2.03	0
***Henan***	66	3283	0.5	9406	2.01	0.005
***Guizhou***	22	1146	0	3484	1.92	0
***Neimenggu***	21	1093	0	2490	1.92	0
***Yunnan***	28	1528	0	4659	1.83	0
***Hebei***	61	3389	0	7288	1.8	0
***Shandong***	75	4329	0	9685	1.73	0
***Guangdong***	109	6992	9.25	10594	1.56	0.0875
***Qinghai***	4	274	0	573	1.46	0
***Xinjiang***	16	1094	0	0	1.46	0
***Xizang***	1	115	0	0	0.87	0

### Content analysis

Among the 292 search results we reviewed, 18 were broken links, six returned a page indicating that the original content had been removed by the website administrator, and 15 webpages were irrelevant to CB suicide (e.g. they were about how to burn pieces of wood to produce charcoal). A total of 253 webpages were retained for inclusion in the content analysis, including 143 (57%) from news media, 90 (36%) from user-generated pages, and 20 (8%) from knowledge hub pages. Some were captured repeatedly by different keywords, and the most frequently captured was the Wikipedia page for CB suicide (appearing eight times).

Although knowledge hub pages (i.e. wiki-type pages and academic papers) accounted for only a small proportion of total search results, all of them contained detailed information of the CB suicide method, whereas 71.3% of the news reports and 51.1% of user-generated content also described the method in detail (*p*<0.001) (Table 3). The results demonstrate that detailed information about the CB method is widely available online and easily accessible through search engines. Attitude toward suicide were associated with the type of information source (*p*<0.001). Of note, user-generated content was more likely to show a pro-suicide attitude (21.1%) than news reports (7.9%) and knowledge hubs (0%). None of the knowledge hub pages encouraged people at risk of suicide to seek help, which was significantly lower than those for the other two sources, although the rates of both were also low (10.5% for news media and 23.3% for user-generated pages) (*p* = 0.004).

**Table 3:** Comparison between the characteristics of search result contents and information source.

		Information source			
*Characteristics of webpages*		News media (N = 143) n (%)	User-generated (N = 90) n (%)	Knowledge hub (N = 20) n (%)	Fisher’s exact test (*p*)
***Described or visualized detailed information of the CB suicide method***	***Yes***	102 (71.3)	46 (51.1)	20 (100)	<0.001
	***No***	41 (28.7)	44 (48.9)	0	
***Attitude toward suicide***	***Anti***	39 (27.3)	29 (32.2)	1 (5.0)	<0.001
	***Pro***	20 (7.9)	19 (21.1)	0	
	***Neutral***	108 (42.7)	2 (2.2)	7 (35.0)	
	***Mixed***	56 (22.1)	40 (44.4)	12 (60.0)	
***Encourage people at suicide risk to seek help***	***Yes***	15 (10.5)	21 (23.3)	0	0.004
	***No***	128 (89.5)	69 (76.7)	20 (100)	

## Discussion

Our data indicated that suicides using the CB method have already occurred in various regions of mainland China, from the coastal region in the east to the inland west, and were reported in 13 out of the 31 provinces or provincial-level-municipalities. There were increasing trends in CB suicides reported by Chinese newspapers and online searches using CB-related keywords. The province-level internet search intensity was correlated with CB suicide rate. Two-thirds of the web links retrieved using the search engine contained detailed information about the CB suicide method, of which 15% showed pro-suicide attitudes, and the majority (86%) did not encourage people to seek help.

The term “charcoal burning,” which originally referred to a method of cooking or heating a room, has now become widely used to refer to a suicide method by general online users. Given that charcoal is easily accessible in mainland China for cooking and heating purposes, especially in rural area, there is a potential risk of further spread and increased use of the method. Our scan of newspapers found that all of the reported cases occurred in cities. This may be due to the fact that rural suicides are seldom reported by the mass media. Accurate death registration data are needed to estimate the media’s reporting bias. Bearing in mind of the possible reporting bias, the result suggests that this new method might be spread by the news press and the Internet, which have higher circulation or penetration rates in cities than rural areas. Similarly, in Taiwan, based on death registration data, CB suicide was found to emerge more prominently in urban than rural areas and such a disproportional rise diminished rural-urban differences in overall suicide rates [27]. In fact, rural suicide rates used to be much higher than urban areas in the 1990s in mainland China but the gap has narrowed in the new century [28]. In addition, despite that the fact that overall suicide rates have been decreasing in China in the past decade, the same trend is not observed in young males [28]. The increase in urbanization and Internet penetration in mainland China could provide a fertile ground for the spread of CB suicide. In this context, the numerous media reports of suicides, especially suicide pacts, using the CB method and the increasing volume of online search activity is a worrying development. This should be a concern for professionals and policy makers involved in suicide prevention and public health. Any increase in CB suicide does not only concern China but would also have an impact on suicide at a global level, given that this populous country accounts for about 15% of suicide deaths worldwide [5].

Celebrity suicide is often found to have significant copycat effects [29]. For example, a recent study found that, following a famous actor’s suicide by CB, the percentage of CB suicides in all suicides in South Korea rose tenfold from 0.7% (84 cases) in 2008 to 7.9% (1,251 cases) in 2011 [30]. Fortunately, in our review of newspaper reports, so far no national level entertainment star used the CB method to kill himself/herself in mainland China. Only one woman, who ended her life by burning charcoal in her car in 2011, was a participant of a talent show and had some media exposure prior to her death. Other than this woman, all of the other cases were ordinary people who were never reported by the media prior to their suicides. However, that woman’s suicide did not receive as much public attention as the three cases that were highlighted in Fig 2. The three high profile cases were reported by the media as associating with certain types of social problems. For example, it was reported that the suicide attempt in case A was influenced by online chat groups. Case B was a suicide pact resulting in the deaths of three adults from the same family. The case was reported as being triggered by unmanageable credit card debts and most of the media coverage focused on what the government should do to regulate issuing credit cards. Case C live broadcasted his suicidal thoughts and acts on an online microblog but received some mocking and indifferent comments, which appeared to cast negative impacts on his mental status. Media reports of this case focused on online bystanders’ attitudes and emphasized online support and suicide prevention. The present study suggests that, even without fuel from celebrity suicide, the CB method is already diffusing in mainland China. In particular, online chat groups and microblog platforms were reported by the media as important channels for spreading this method.

National or even international intervention strategies are needed to constrain the further spreading of the CB method in China. A national surveillance system should be established to monitor the spread of any new suicide method in China. According to media reports, CB suicides often took place in hotels. Therefore, hotel staff should be engaged as gatekeepers to watch for the possible incidence of CB suicide and/or to oversee the installation of carbon monoxide detectors. Monitoring the information available via various media platforms can also be an alternative to alert suicide prevention professionals to new trends in suicide methods [7].

Our results also suggest an interaction between traditional news media reports and online searching at both national and provincial levels. The findings are consistent with another recent study on traditional media reports and online search activities [14]. A particular example identified in our study is the sharp increase in online searches about CB suicide after a young man broadcast his suicide live on social media and this was reported by traditional news sources (case C in Fig 2). The case demonstrated a chain of information diffusion, from an individual’s personal microblogging, to its spread on social media, to reports in the traditional news media, and finally becoming retrievable by online users, anytime and anywhere. The influence of this diffusion chain seems to accumulate and last rather than fade away. Studies show that prominent media representations of new suicide methods can lead to significant copycat effects, especially among young people [12]. Further studies may try to investigate whether a diffusion chain combining both traditional and social media can magnify this effect.

To counterbalance possible copycat effects, we need to engage diverse stakeholders involved in the diffusion chain, including the administrators or managers of social media sites, journalists in the traditional news media, managers of search engine companies, and users as a whole. It will be strategically important to introduce the WHO media guidelines for suicide reporting to media professionals in mainland China and also update the guidelines for use in the online environment. Media professionals in mainland China, including those working for either the traditional mass or online media, need to be reminded about how to present news about suicide responsibly and in particular to avoid detailed descriptions of methods [31,32]. Because restricting access to the means of suicide has been found to be an effective strategy in prevention [33]. When a new method emerges, we must try to prevent and/or limit its spread not only physically, but also cognitively via the media [15, 34].

The online media is playing a significant role in spreading information about suicide, and everything else, as the Internet penetration rate in China reached 46.9% by mid-2014 and is still increasing exponentially [35]. Online service providers in western countries, such as Twitter, Google, and Facebook, have put in place policies to encourage and facilitate their users to seek help when they are at risk of suicide [36–39]. Mainland Chinese online service providers should also consider displaying helpline information whenever their users search, discuss, or post information relating to suicide, and assigning moderators for discussion groups relating to suicide who can encourage people to seek help.

It would be challenging, if not impossible, to remove all the information about CB suicide from the Internet. Nonetheless, strengthening legal or policy regulation of pro-suicide information is still a necessary element in prevention. In one individual case, an Internet service provider in China that allowed pro-suicide information to be posted online was prosecuted [39]. However, China practices the civil law system, which means the individual ruling has limited impact on future cases. Instead, codified statutes and ordinances dominate how to decide a case at hand. News or other online information that describes details of suicide methods, encourages suicide, or facilitates suicide is not defined as illegal under Chinese law nor as harmful by any of the regulatory or administrative guidelines. The only exception is that Chinese courts can prosecute cult organizations for intentionally organizing, encouraging, or helping their followers to kill or injure themselves [40]. Many countries, such as Japan, Brazil, Italy, England, and Switzerland have clearly defined abetting or aiding suicide as illegal [41]. It is also of significant public interest for Chinese citizens and legal professionals to discuss how to regulate pro-suicide information on the Internet.

It is noteworthy that knowledge hub pages, including all of the 18 wiki-type webpages and 2 webpages of academic publications, contain detailed descriptions of suicide methods, but none of them encourages people to seek help. In addition, among the 20 hub pages, only one page of academic publication showed clear attitude of suicide prevention. The two academic publications, one reported a charcoal burning case in a forensic journal and the other reviewed how to conduct crime scene investigation for CB suicide cases. Such results show that Internet hubs for neutral knowledge exchange can lead to undesirable consequences if they only describe methods in detail without balancing this with preventive messages. Scholars and suicide prevention professionals should contribute more actively to wiki-type websites by editing suicide-related content and should also be more sensitive when publishing their work. In the Internet era, academic publications are not read by scholars alone, but can be accessed by the general public. Contextual information, including preventive messages, should be included in such publications so that lay readers can be guided to sources of help.

The study has some limitations. The news reports were collected from the WiseNews database, which may not include all newspapers in mainland China. Furthermore, one previous study shows that mainland Chinese media do not report suicide news as frequently as in Hong Kong or Taiwan [24]. Hence, it is likely that we have underestimated the incidence of CB suicides. In addition, the online search data were secondary data extracted from Baidu and may have been processed by the Baidu algorithms. If Baidu agrees to share raw data on search trends with researchers or/and suicide prevention organizations, the study could be improved and a more comprehensive surveillance system could be established. Nevertheless, we have exhaustively examined relevant data that can be publicly accessed. More studies should be conducted to investigate further the probability and mechanisms of individuals being exposed to, and even “infected” by, pro-suicide information online [42].

## Conclusion

The study provides an overall picture of the spreading trend of CB suicide in mainland China from a media perspective. It demonstrates that this suicide method has been introduced to mainland China by social media and has occurred in many regions of the country. We are aware that media reports may not cover all suicide incidences, and that online users do not represent all of the Chinese population. Nevertheless, off- and online media content shows that information about this suicide method is readily accessible and numerous Chinese users have been searching for it. Therefore, the prevention of CB suicide should be included on the agenda of suicide prevention in mainland China, and also deserves international concern. Surveillance systems for suicide incidence, with CB as a specific subcategory of method, should be established in mainland China. Careful consideration should be given by the government and policymakers to introducing regulatory control of risky information available online. Chinese online service providers should learn from the experiences of their overseas counterparts and develop functions and services to encourage and facilitate their users to seek help when feeling suicidal. In order to maintain the decreasing trend of the suicide rate in China over the past decade, engaging vulnerable people via various media channels is a necessary and promising step, if it can be properly implemented and monitored.

## References

[1] Yip PS, Kwok SS, Chen F, Xu X, Chen YY. A study on the mutual causation of suicide reporting and suicide incidences. J Affect Disord. 2013; 148: 98–103. 10.1016/j.jad.2012.11.056 23260382

[2] Lee DT, Chan KP, Lee S and Yip PS. Burning charcoal: a novel and contagious method of suicide in Asia. Arch Gen Psychiatry. 2002; 59: 293–294. 11879176 10.1001/archpsyc.59.3.293

[3] Chang SS, Chen YY, Yip PS, Lee WJ, Hagihara A, Gunnell D. Regional changes in charcoal-burning suicide rates in East/Southeast Asia from 1995 to 2011: a time trend analysis. PloS Medicine. 2014; 11 (4): e1001622 10.1371/journal.pmed.1001622 24691071 PMC3972087

[4] Thomas K, Chang S-S, Gunnell D. Suicide epidemics: the impact of newly emerging methods on overall suicide rates—A time trends study. BMC Public Health. 2011; 11:314 21569569 10.1186/1471-2458-11-314 PMC3112128

[5] World Health Organization. Preventing Suicide: A Global Imperative. 2014.

[6] Chen Y-Y, Bennewith O, Hawton K, Simkin S, Cooper J, Kapur N, et al Suicide by burning barbecue charcoal in England. J Public Health. 2013; 35: 223–227. 10.1093/pubmed/fds095 23179241

[7] Cheng Q, Chang SS, Yip PS. Opportunities and challenges of online data collection for suicide prevention. Lancet. 2012; 379: e53–54. 10.1016/S0140-6736(12)60856-3 22633035

[8] Zeng D, Huang BH, Huang XH. Burning charcoal in an enclosed space 1 case. J Forensic Med. 2008; 24: 392–393.

[9] Tian L, Chen Z. Burning charcoal to carbon monoxide poisoning suicide 3 cases. J Forensic Med. 2009; 25: 306–307.

[10] Li F, Chan HC, Liu S, Jia H, Li H, Hu Y, et al Carbon monoxide poisoning as a cause of death in Wuhan, China: A retrospective six-year epidemiological study (2009–2014). 2015; 253: 112–118. 10.1016/j.forsciint.2015.06.007 26115227

[11] Chen Y-Y, Chen F, Gunnell D, Yip PSF. The impact of media reporting on the emergence of charcoal burning suicide in Taiwan. PLoS ONE. 2013; 8: e55000 10.1371/journal.pone.0055000 23383027 PMC3559477

[12] Hagihara A, Abe T, Omagari M, Motoi M, Nabeshima Y. The impact of newspaper reporting of hydrogen sulfide suicide on imitative suicide attempts in Japan. Soc Psychiatry Psychiatr Epidemiol. 2014; 49: 221–229. 10.1007/s00127-013-0741-8 23851704

[13] Hagihara A, Miyazaki S, Abe T. Internet suicide searches and the incidence of suicide in young people in Japan. Eur Arch Psychiatry Clin Neurosci. 2012; 262: 39–46. 10.1007/s00406-011-0212-8 21505949

[14] Chang S-S, Kwok S, Cheng Q, Yip PF, Chen Y-Y. The association of trends in charcoal-burning suicide with Google search and newspaper reporting in Taiwan: a time series analysis. Soc Psychiatry Psychiatr Epidemiol. 2015 4 10 10.1007/s00127-015-1057-7 25859754

[15] Biddle L, Gunnell D, Owen-Smith A, Potokar J, Longson D, Hawton K, et al Information sources used by the suicidal to inform choice of method. J Affect Disord. 2012; 136: 702–709. 10.1016/j.jad.2011.10.004 22093678

[16] Chan KP, Yip PS, Au J, Lee DT. Charcoal-burning suicide in post-transition Hong Kong. Br J Psychiatry. 2005; 186: 67–73. 15630126 10.1192/bjp.186.1.67

[17] Tsai CW, Gunnell D, Chou YH, Kuo CJ, Lee MB, Chen Y-Y. Why do people choose charcoal burning as a method of suicide? An interview based study of survivors in Taiwan. J Affect Disord. 2011; 131: 402–407. 10.1016/j.jad.2010.12.013 21236495

[18] National Bureau of Statistics of the People's Republic of China. The 2010 Population Census of The People's Republic of China. Beijing. 2010.

[19] China Internet Network Information Center. The 33rd Statistical Report on Internet Development in China (in Chinese). 2014.

[20] Incitez China. China search engine market share in Q3 2014. China Internet Watch. 2014.

[21] Jenks GF. The data model concept in statistical mapping. International Yearbook of Cartography. 1967; 7.1: 186–190.

[22] Cheng Q, Fu KW, Yip PSF. A comparative study of online suicide-related information in Chinese and English. J Clin Pscyhiatry. 2011; 72: 313–319. 10.4088/JCP.09m05440blu 20868633

[23] Biddle L, Donovan J, Hawton K, Kapur N, Gunnell D. Suicide and the internet. BMJ (Clinical research ed). 2008; 336: 800–802. 10.1136/bmj.39525.442674.AD PMC2292278 18403541

[24] Fu KW, Chan YY, Yip PS. Newspaper reporting of suicides in Hong Kong, Taiwan and Guangzhou: compliance with WHO media guidelines and epidemiological comparisons. J Epidemiol Community Health. 2011; 65: 928–933. 10.1136/jech.2009.105650 20889589

[25] China Daily. Alarming 'Credit card slavery'. China Daily. Beijing. 2014 Nov 25. Retrieved from http://www.chinadaily.com.cn/opinion/2014-11/25/content_18971253.htm.

[26] Xinhua News Agency. Live online suicide raises concerns in China. Beijing: Xinhua News Agency. 2014 Dec 1st. Retrieved from http://news.xinhuanet.com/english/china/2014-12/01/c_133826060.htm.

[27] Chang S-S, Gunnell D, Wheeler BW, Yip PS, Sterne JA. The evolution of the epidemic of charcoal-burning suicide in Taiwan: A spatial and temporal analysis. PloS Medicine. 2010; 7: e1000212 10.1371/journal.pmed.1000212 20052273 PMC2794367

[28] Wang C-W, Chan CL, Yip PSF. Suicide rates in China from 2002 to 2011: An update. Soc Psychiatry Psychiatr Epidemiol. 2014; 49(6):929–41. 10.1007/s00127-013-0789-5 24240568

[29] Niederkrotenthaler T, Fu K-W, Yip PSF, Fong DYT, Stack S, Cheng Q, et al Changes in suicide rates following media reports on celebrity suicide: a meta-analysis. J Epidemiol Community Health. 2012; 66:1037–1042. 10.1136/jech-2011-200707 22523342

[30] Ji N-J, Hong Y-P, Stack SJ, Lee W-Y. Trends and risk factors of the epidemic of charcoal burning suicide in a recent decade among Korean people. J Korean Med Sci. 2014; 29(8):1174–1177. 10.3346/jkms.2014.29.8.1174 25120332 PMC4129214

[31] Cheng Q, Fu KW, Caine E, Yip PS. Why do we report suicides and how can we facilitate suicide prevention efforts? Perspectives of Hong Kong media professionals. Crisis. 2014; 35: 74–81. 10.1027/0227-5910/a000241 24322824 PMC4150930

[32] Bohanna I, Wang X. Media guidelines for the responsible reporting of suicide: a review of effectiveness. Crisis. 2012; 33: 190–198. 10.1027/0227-5910/a000137 22713977

[33] Yip PS, Caine E, Yousuf S, Chang SS, Wu KC, Chen Y-Y. Means restriction for suicide prevention. Lancet. 2012; 379: 2393–2399. 10.1016/S0140-6736(12)60521-2 22726520 PMC6191653

[34] Florentine JB, Crane C. Suicide prevention by limiting access to methods: a review of theory and practice. Soc Sci Med. 2010; 70: 1626–1632. 10.1016/j.socscimed.2010.01.029 20207465

[35] China Internet Network Information Center. Statistic Report on Internet Development in China. Beijing. 2014.

[36] Twitter Help Center. Dealing with self-harm and suicide. Retrieved from https://support.twitter.com/articles/20170313-dealing-with-self-harm-and-suicide

[37] Facebook Help Center. Suicide Prevention. Retrieved from https://www.facebook.com/help/594991777257121/

[38] Milian M. Facebook, Google refer suicidal people to help lines. CNN. 2011 Dec 13. Retrieved from http://edition.cnn.com/2011/12/13/tech/web/facebook-google-suicide/

[39] Cheng Q. Are internet service providers responsible for online suicide pacts? BMJ. 2012; 344:d2113 10.1136/bmj.d2113 22505035

[40] Supreme People's Court of the People's Republic of China and Supreme People's Procuratorate of the People's Republic of China. Explanations on legal issues relating to criminal activities that are organized through cult organizations II (in Chinese). (Jun 4, 2001).

[41] Chen X. Study on the nature of abetting or aiding other' suicide: A case study on Shao Jianguo case (in Chinese). Zhejiang Soc Sci. 2004; 6:71–78.

[42] Cheng Q, Li H, Silenzio V, Caine ED. Suicide contagion: A systematic review of definitions and research utility. PLoS ONE. 2014; 9: e108724 10.1371/journal.pone.0108724 25259604 PMC4178222

